# Comparative Interaction Mechanisms and Solution Behavior of Cowhide Collagen with Xanthan Gum, Gellan Gum, and Chitosan Under Variable Environmental Conditions

**DOI:** 10.3390/foods14234107

**Published:** 2025-11-29

**Authors:** Kaiyuan Li, Zhuangzhuang Wang, Ang Ru, Ke Wang, Wenming Cui, Chaozhi Zhu, Gaiming Zhao, Jiangang Hao

**Affiliations:** 1 Henan Key Lab of Meat Processing and Quality Safety Control, Henan Agricultural University, Zhengzhou 450002, China; kyli0050@163.com (K.L.);; 2 College of Food Science and Technology, Henan Agricultural University, Zhengzhou 450002, China; 3 National Beef Cattle and Yak Industry Technology System Wulagai Comprehensive Test Station, Neimenggu 026321, China

**Keywords:** cowhide collagen, protein–polysaccharide interaction, electrostatic and hydrogen bonding mechanisms, rheological behavior, structural characterization, sustainable utilization of animal by-products

## Abstract

Cowhide collagen (CC) is a valuable by-product of the meat industry with promising applications in food systems; however, its poor viscosity and limited stability restrict its practical use. This study systematically investigated the interactions between CC and three representative polysaccharides—xanthan gum (XG), gellan gum (GG), and chitosan (CS)—under varying concentrations, pH, and ionic strengths. The physicochemical behaviors of the composite systems were evaluated through turbidity, fluorescence spectroscopy, Fourier transform infrared (FTIR) analysis, and rheological measurements. The experimental results revealed a pronounced increase in the turbidity of the GG–CC system, rising from approximately 0.18 ± 0.01 to 2.14 ± 0.01 as the polysaccharide concentration increased, with maximum values exceeding 2.0 under several conditions. Similarly, both the apparent viscosity and turbidity of the other two PS–CC composite systems exhibited a marked and progressive enhancement with increasing polysaccharide content. FTIR spectra confirmed strengthened O–H stretching and amide I shifts, indicating intensified hydrogen bonding and electrostatic interactions. High NaCl levels disrupted the protein hydration shell, modifying fluorescence intensity and peak sharpness. XG–CC and GG–CC composites exhibited similar behaviors, while CS–CC systems showed opposite pH-dependent trends due to cationic–cationic repulsion. Overall, polysaccharide type and concentration exerted stronger effects on CC structure and rheology than environmental factors. These results clarify how polysaccharide type and environmental factors modulate collagen–polysaccharide interactions and provide practical guidance for selecting polysaccharides and processing conditions to tailor the rheological and stability properties of collagen-based food ingredients.

## 1. Introduction

Collagen, the most abundant structural protein in mammals, is a fundamental component of connective tissues such as skin, tendon, and bone [1]. Owing to its characteristic triple-helical structure, collagen exhibits remarkable biocompatibility, film-forming capacity, and nutritional value, making it a versatile biomaterial with wide applications in the food, pharmaceutical, and biomedical industries [2]. As a renewable and biodegradable protein, collagen has drawn increasing attention in recent years for its potential to support sustainable material development and functional food innovation. Collagen derived from different animal sources exhibits distinct physicochemical properties that determine their specific functional roles and applications. Porcine skin collagen, known for its high solubility and excellent biocompatibility, is mainly applied in biomedical and cosmetic products such as wound dressings and tissue regeneration scaffolds [3,4]. Bovine bone collagen, characterized by a high degree of crosslinking and close association with hydroxyapatite minerals, possesses high rigidity and is thus suitable for bone tissue engineering and mineralized composite development [5,6]. In contrast, cowhide collagen (CC) exhibits moderate crosslinking, good solubility, and superior adaptability to changes in pH and ionic strength, making it particularly suitable for food applications such as colloids, gels, and emulsions with controlled rheological and stability characteristics [7,8]. CC is also an abundant, low-cost by-product of the meat processing industry. Its valorization not only promotes the sustainable utilization of animal by-products but also contributes to environmental protection and economic efficiency [9]. Therefore, developing methods to extract and enhance the functionality of CC is of considerable industrial and ecological importance.

Recent advancements in extraction technologies—such as ultrasound-assisted, high-pressure-assisted, and microwave-assisted enzymatic methods—have markedly improved the yield, purity, and structural integrity of CC [10,11]. These innovative processes preserve the native triple-helical structure of collagen while reducing processing time and energy consumption [12]. Although such methods enhance CC recovery and quality, its native form still suffers from low viscosity, poor rheological behavior, and limited thermal stability, and it tends to aggregate or precipitate under fluctuating environmental conditions. These shortcomings significantly restrict its direct use in emulsified, gelled, and colloidal food systems, as well as in biomaterial applications. Consequently, forming composite systems with other macromolecules has become a promising strategy to overcome these functional limitations and broaden CC’s application potential.

Among various modifiers, polysaccharides have attracted significant interest due to their natural origin, hydrophilicity, and diverse functional properties. They interact with proteins through hydrogen bonding, electrostatic attraction, and hydrophobic forces, thereby influencing the physicochemical, structural, and rheological behaviors of composite systems [13]. Polysaccharide-modified collagen complexes offer unique advantages in food and biomaterial applications. For example, compared with synthetic polymers, polysaccharides offer a cleaner label and superior biocompatibility [14], and unlike some protein–polyphenol complexes that may introduce undesirable astringency or color changes, polysaccharide–collagen interactions can modulate physical properties such as viscosity and stability to achieve precise control over texture and functionality without significantly altering sensory attributes [15]. Polysaccharides are widely used in food processing as thickeners, stabilizers, emulsifiers, and gelling agents, and their ability to interact with proteins is a key determinant of product texture and stability [13]. The nature and strength of protein–polysaccharide interactions depend on the structural characteristics and charge distribution of both biopolymers, as well as on environmental factors such as pH, ionic strength, and concentration [16,17]. Previous studies have explored such interactions in model systems, including fish gelatin–xanthan, casein–gellan, and soy protein–chitosan complexes, and confirmed that the type and charge of polysaccharides, together with environmental parameters, play decisive roles in the formation and stability of complexes [18,19,20]. However, studies investigating how polysaccharide concentration, pH, and ionic strength collectively influence the molecular interactions and macroscopic behaviors of collagen—particularly type I collagen derived from cowhide—remain limited. Bridging this knowledge gap will facilitate the systematic design of collagen-based materials with optimized rheological, structural, and stability properties, thereby providing new strategies for improving texture, stability, and functionality in food applications.

Previous research has demonstrated that electrostatic forces dominate when proteins and polysaccharides carry opposite charges, leading to complex coacervation or soluble complex formation depending on charge balance [21]. Conversely, when both macromolecules have similar charges, hydrogen bonding and hydrophobic interactions become more prominent [22]. These interactions not only modify the microstructure of composite systems but also govern macroscopic properties such as turbidity, viscosity, and gel strength. Therefore, understanding the mechanisms underlying protein–polysaccharide interactions is crucial for tailoring the structural and functional attributes of composite food materials [23,24]. Among food-grade polysaccharides, xanthan gum (XG), gellan gum (GG), and chitosan (CS) are typical representatives exhibiting distinct charge types and molecular structures [25]. XG and GG are anionic polysaccharides characterized by high viscosity, shear-thinning behavior, and excellent stability across a broad range of pH and temperature conditions, whereas CS is a cationic polysaccharide derived from chitin, notable for its strong film-forming ability, antimicrobial activity, and superior biocompatibility [26]. These contrasting physicochemical characteristics provide an ideal framework for systematically examining how electrostatic and hydrogen-bonding interactions with collagen are modulated under different environmental conditions. Understanding these differences is crucial for elucidating the mechanisms governing collagen–polysaccharide associations and for guiding the rational design of functional food and biomaterial systems with optimized structural and rheological properties.

In this study, we systematically investigated the interaction mechanisms and solution behaviors of cowhide collagen with XG, GG, and CS under varying polysaccharide concentrations, pH, and ionic strengths. Through integrated spectroscopic, rheological, and microstructural analyses, this research provides new insights into how electrostatic and hydrogen bonding interactions determine the physicochemical performance of collagen–polysaccharide composites, thereby offering a scientific foundation for the development of texture-modified foods, functional emulsions, and bio-based packaging materials.

## 2. Materials and Methods

### 2.1. Materials

The cowhide (after dehairing and deashing) was supplied from Henan HengDu food Co., Ltd. (Zhumadian, China), and it was divided into small pieces, placed in self-sealing bags, and stored at −20 °C. XG, GG, and CS were of food grade and obtained from Henan WanBang chemical technology Co., Ltd. (Zhengzhou, China). Pepsin (CAS 901-75-6) from porcine stomach with an activity of 2500 U/mg was purchased from Beijing Solarbio science and technology Co., Ltd. (Beijing, China). Other analytical grade chemical reagents were purchased from Beijing Sinopharm chemical reagent Co., Ltd. (Beijing, China).

### 2.2. Extraction of CC from Cowhide

Extraction process of CC: Fresh cowhide → Defatting → removal of non-collagen protein → Cold storage → Thawing → High-speed chopping → Pretreatment → Enzymatic extraction/Ultrasonic-assisted enzymatic extraction → Salt precipitation → Dialysis → Freeze-drying → Collagen (Supplementary Materials). Fresh cowhide collagen was defatted and non-collagen protein was removed by sequential treatment with 5% Na_2_CO_3_ and NaCl solutions, followed by extensive washing until no residual chloride ions were detected. The cleaned hide fragments were cut into 1 × 1 cm pieces and stored at −20 °C. After thawing, the samples were subjected to acid swelling in citric acid monohydrate (pH 2.0) at 0–4 °C for 24 h and homogenized at 10,000 rpm for 5 min. Bovine collagen was extracted using two enzymatic approaches: a conventional enzymatic extraction method (EEM) and an ultrasonic-assisted enzymatic extraction method (UEEM). In the EEM process, the homogenized bovine hide was hydrolyzed with pepsin (105 U/g collagen) at 37 °C for 8 h. The UEEM process applied ultrasonic assistance to the EEM process, in which the homogenate was mixed with pepsin and treated with ultrasound at 161 W for 64 min, followed by enzymatic hydrolysis at 37 °C, with the total hydrolysis time controlled for 8 h. Following hydrolysis, the supernatant obtained by centrifugation was subjected to salt precipitation using 0.9 mol/L NaCl at 4 °C, and the precipitation was dissolved in 0.5 mol/L citric acid. Dialysis against citric acid and distilled water for 24–48 h removed impurities, after which the solution was freeze-dried to yield a white, porous collagen product. The resulting samples were designated as EE (conventional) and UEE (ultrasonic assisted).

### 2.3. Preparation of Polysaccharide–Cowhide Collagen (PS–CC)

The CC solution (40 mg/mL) was prepared using a high-speed disperser (T25, IKA, Staufen, Germany). XG (0.05%, 0.1%, 0.2%, 0.3%, 0.4% (*w*/*w*)), GG (0.1%, 0.2%, 0.3%, 0.4%, 0.5% (*w*/*w*)) or CS (0.2%, 0.4%, 0.6%, 0.8%, 1.0% (*w*/*w*)) were added, respectively, to make the composite reach its optimal range at room temperature. The concentration range of polysaccharides was determined based on preliminary solubility and viscosity tests combined with their typical usage levels in food formulations. This range was selected to ensure homogeneity and measurable interactions with collagen while avoiding phase separation or excessively high viscosity that could interfere with the analyses. After homogeneous dispersion, the samples were divided into two parts. One group of the XG-CC, GG-CC, and CS-CC composites was adjusted to pH 4, 5, 6, 7, and 8. The other group was combined with 50, 100, 200, 300, and 400 mM NaCl.

### 2.4. Characterization of CC

To ensure the consistency of the subsequent polysaccharide–collagen interaction experiments, the collagen obtained from the optimized UEEM was selected as the model substrate. However, both the EEM and UEEM samples underwent parallel characterization, including SDS–PAGE, FTIR, and amino acid composition analyses, to verify their collagen type, purity, and structural integrity.

#### 2.4.1. Determination of CC by SDS–PAGE

Sodium dodecyl sulfate–polyacrylamide gel electrophoresis (SDS–PAGE) was performed according to the method of Sahni et al. [27] with minor modifications. Collagen samples were dissolved to a concentration of 4 mg/mL. The separating and stacking gels were prepared at concentrations of 8% and 5%, respectively. The sample solution was mixed with loading buffer (1:1, *v*/*v*), heated in a boiling water bath for 5 min, and briefly vortexed before loading. Electrophoresis was conducted until the tracking dye reached the bottom of the separating gel, followed by staining with Coomassie Brilliant Blue R-250 (Solarbio science and technology Co., Ltd., Beijing, China) and destaining with a methanol/acetic acid solution until clear protein bands appeared. The gel was then imaged and analyzed using a gel documentation system.

#### 2.4.2. Determination of CC by Fourier Transform Infrared Spectroscopy

Fourier transform infrared (FTIR) spectroscopy (Bruker Co., Ltd., Ettlingen, Germany) was conducted according to the method of Zhu et al. [28] using an attenuated total reflectance (ATR) accessory. Each sample was scanned from 400 to 4000 cm^−1^ at a resolution of 4 cm^−1^, and the final spectrum was obtained by averaging 32 scans. Changes in the characteristic absorption peaks were recorded to evaluate the structural features of the collagen samples, including the presence of the triple-helix conformation and functional group integrity. 

#### 2.4.3. Analysis of Amino Acid Composition of CC

The amino acid composition was determined according to the Chinese National Standard GB 5009.124-2016 [29]. Approximately 50 mg of collagen sample was accurately weighed into a hydrolysis tube and mixed with hydrochloric acid. The solution was flushed with nitrogen for 10 min and sealed under heat. Hydrolysis was performed at 110 °C for 24 h. After hydrolysis, the filtrate pH was adjusted to approximately 9.0 and the volume was brought up to 25 mL with deionized water. The resulting solution was derivatized before analysis and filtered through a 0.45 μm membrane. The amino acid composition (ACQUITY UPLC Amino Acid Analysis Solution, Waters Corporation, USA) was determined using an automatic amino acid analyzer under standard operating parameters.

### 2.5. Physical Appearance of PS–CC Composite

The sample was taken out of a 4 °C refrigerator and stabilized at 25 °C for 2 h. A total of 3 mL of fully hydrated XG-CC composites of different kinds were transferred into thread bottles, any existing bubbles were removed, and the appearance of the composite was observed from a macro perspective.

### 2.6. Turbidity of PS–CC Composite

Samples were equilibrated at room temperature for 2 h after removal from storage at 4 °C and then diluted five-fold. Next, the absorbance was monitored at 600 nm using a UV-2600 spectrophotometer (Shimadzu, Tokyo, Japan). Turbidity was defined as absorbance, and the turbidity value was corrected against blank deionized water.

### 2.7. Viscosity of PS–CC Composite

Using a TA-ARES-G2 rotary rheometer (TA Company, New Castle, DE, USA), testing was performed using a 40 mm plate with a test spacing of 450 μm. The temperature was set to 25 ± 0.5 °C, the ramp mode was selected, the shear rate range was set to 0~300 s^−1^, the test result was based on the viscosity change during the change in shear rate, and the test was repeated 3 times.

### 2.8. Determination of Fluorescence Spectrum of PS–CC Composite

Endogenous fluorescence was measured using a F-4600 fluorescence spectrophotometer (Shimadzu, Japan), and the samples were placed in a four-sided light-transmitting sample cell with a 1 cm optical path. The excitation wavelength was set at 280 nm during measurement, the emission wavelength range at 200–450 nm, and the excitation and emission wavelength slit were set at 10 nm. The experimental values were repeated three times, and the average value of the three scanning results was taken.

### 2.9. Determination of PS–CC Composite by Fourier Transform Infrared Spectroscopy

The composite samples were ground into powder, sieved, and mixed with KBr at a ratio of 1:100 for determination at 25 °C. The spectra were collected in the range of 400–4000 cm^−1^ with a resolution of 4 cm^−1^ and averaged over 32 scans using a TEN-SOR Ⅱ Fourier infrared spectrometer (Bruker Co., Ltd., Ettlingen, Germany). The spectral data underwent analysis using Peakfit 4.11 software (Systat Software Inc., San Jose, CA, USA).

### 2.10. Statistical Analysis

Tests were repeated three times, and the samples were measured in triplicate for each analysis. All results were expressed as means ± standard deviations (SD). Statistical calculations were performed with SPSS 27.0 (SPSS 27.0, IBM, Chicago, IL, USA) One-way analysis of variance was employed to determine the significance of the main effects by means of a Duncan’s multiple range test with a significance level set at *p* < 0.05. All figures were prepared with Origin Pro 9.2 (Origin Lab Co., Northampton, MA, USA).

## 3. Results and Discussion

### 3.1. Structural Characterization of CC

**Figure 1:**
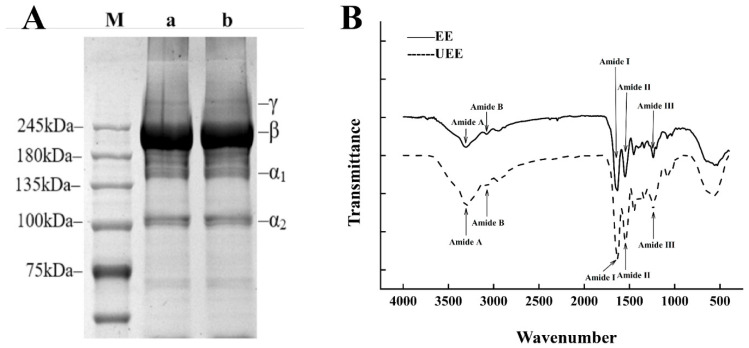
SDS-PAGE (**A**) and FTIR spectrogram (**B**) of both EE and UEE.

The collagen characterization results revealed that CC extracted by both EEM and UEEM exhibited comparable structural and compositional properties characteristic of type I collagen. SDS–PAGE analysis showed nearly identical electrophoretic patterns, with distinct γ-, β-, α_1_-, and α_2_-chain bands corresponding to molecular weights above 245 kDa, near 200 kDa, 135 kDa, and 110 kDa, respectively, confirming the preservation of intact collagen subunits (Figure 1A). FTIR spectra further supported this finding, showing consistent amide A (≈3300 cm^−1^), amide B (≈3080 cm^−1^), amide I (≈1640 cm^−1^), amide II (≈1545 cm^−1^), and amide III (≈1235 cm^−1^) bands, indicative of the typical triple-helical conformation of type I collagen (Figure 1B). Amino acid analysis revealed no significant compositional differences between the two extraction methods, with glycine and proline as the dominant residues, reflecting the characteristic (Gly–X–Y) repeating motif of collagen (Table 1). Collectively, these findings demonstrate that both EE and UEE produced structurally intact and compositionally consistent type I collagen, while the UEEM provided enhanced extraction efficiency and superior functional performance (Supplementary Materials).

These structural characteristics are in good agreement with previous studies reporting type I collagen extracted from bovine and porcine sources by enzymatic or ultrasound-assisted methods, which also exhibited typical α_1_- and α_2_-chain bands in SDS–PAGE and well-preserved amide I–III peaks in FTIR spectra [30]. This consistency confirms that the collagen obtained in the present study retained its native triple-helical structure and type I characteristics, validating its suitability as a substrate for subsequent polysaccharide–collagen interaction analysis.

### 3.2. Effect of pH on the Physical Appearance and Turbidity of PS–CC Composite

The turbidity of the composite systems revealed distinct interaction patterns for each polysaccharide, primarily governed by electrostatic forces.

**Figure 2:**
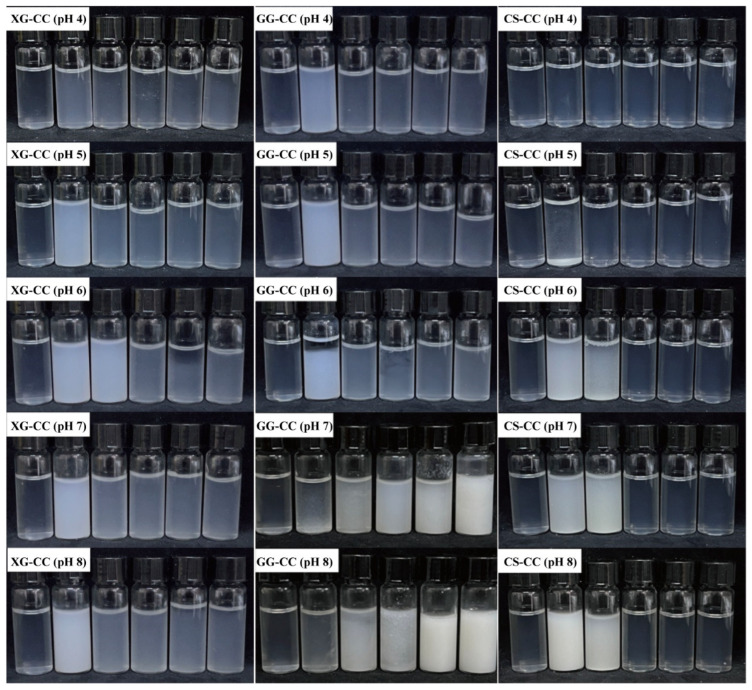
The effects of different pH values on the physical appearance of XG-CC, GG-CC, and CS-CC composites. There are six bottles in each subfigure, which were the control group and the XG-CC, GG-CC, or CS-CC composites with five polysaccharide concentrations, increasing in turn.

For the anionic polysaccharides (XG and GG), the highest turbidity was consistently observed at a low pH (Figure 2 and Table 2). This can be attributed to the strong electrostatic attraction between the negatively charged polysaccharides and the positively charged cowhide collagen (CC) under acidic conditions (pH < pI~6.0 of CC), which promotes the formation of insoluble complexes and aggregates [31]. For XG-CC, turbidity decreased sharply as the pH increased from 4 to 6–7, minimizing near the isoelectric point of CC where the protein’s net charge approaches zero, thereby weakening the electrostatic driving forces. A similar trend was observed for GG-CC, where turbidity remained high at low pH but stabilized at higher pH levels (≥5). At these higher pH values, the weakening electrostatic interaction was likely counterbalanced by a significant increase in system viscosity, which can hinder aggregate growth and precipitation [32]. Furthermore, the persistence of turbidity under net-repulsive electrostatic conditions suggests that hydrophobic interactions may also contribute to sustaining the composite structure.

In contrast, the cationic polysaccharide CS exhibited a fundamentally different, pH-dependent behavior driven by electrostatic repulsion. At low CS concentrations (≤0.4%), turbidity remained uniformly low across the entire pH range, as repulsive forces between the similarly charged CS and CC prevented significant aggregation. However, at higher CS concentrations (≥0.6%), a dramatic increase in turbidity occurred, but only within a specific pH window of 4–5. This phenomenon is best explained by charge neutralization: as the pH approaches the isoelectric point of CC (pI~6.0), the net positive charge on the protein decreases sufficiently to allow the cationic CS to bridge and neutralize adjacent protein molecules, leading to extensive aggregation and phase separation [33]. When the pH increases beyond this window (pH > 6), the net negative charge on CC increases, reinstating strong electrostatic repulsion with CS and dispersing the complexes, which consequently leads to a sharp drop in turbidity. Although statistical analysis indicated significant differences (*p* < 0.05) among some pH points at 0.4% CS concentration (Table 2), the absolute turbidity values were all very low (ranging from 0.10 to 0.22) and these minor variations are not considered to be of practical relevance. The key practical finding is the consistent lack of substantial turbidity development across the entire pH range at this low CS concentration, indicating an absence of major aggregation.

In summary, a comparative analysis of the turbidity clearly delineates the unique interaction paradigm for each polysaccharide type. The systems segregate not by the magnitude of turbidity, but by the underlying electrostatic mechanism responsible for it. XG-CC and GG-CC, as anionic polysaccharides, both rely on electrostatic attraction to drive aggregation at low pH. Their divergence at higher pH highlights GG’s superior capacity to sustain complexes, potentially via viscosity and hydrophobic forces. Conversely, the CS-CC system operates under a fundamentally different rule set, where aggregation is not driven by attraction but by the suppression of repulsion via charge neutralization near CC’s pI. Thus, the anionic polysaccharides are defined by an “attraction-dispersion” model, while the cationic CS is defined by a “repulsion-neutralization-dispersion” model. This fundamental distinction in governing mechanisms elegantly explains the observed diversity in pH-dependent solution behavior.

### 3.3. Effect of pH on the Apparent Viscosity of PS–CC Composite

**Figure 3:**
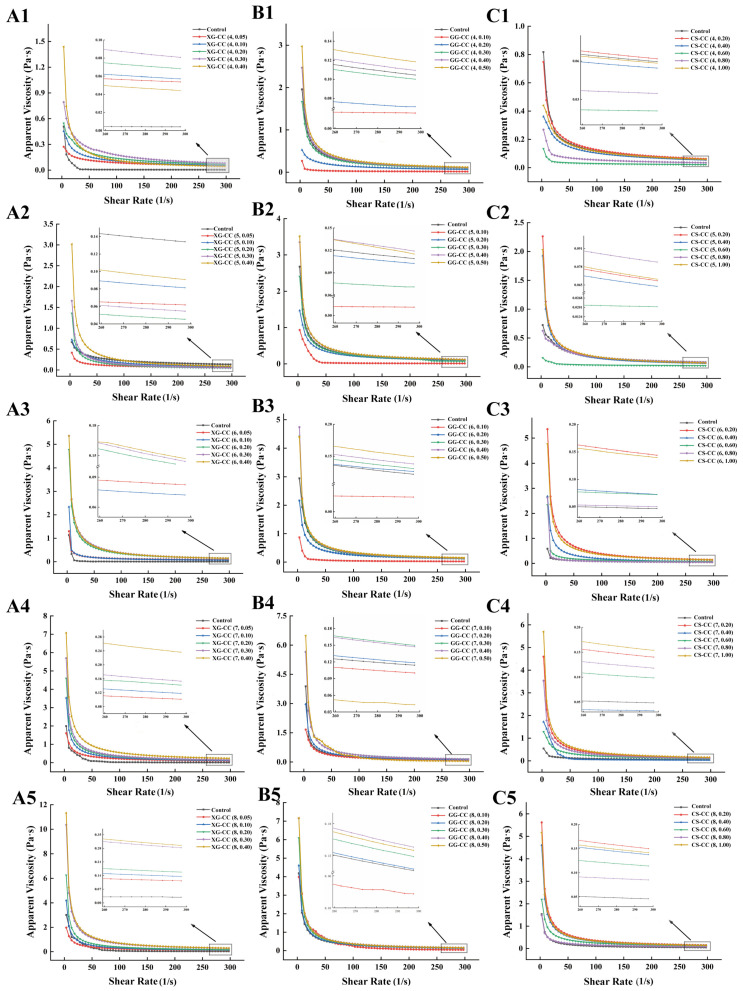
The effects of different pH values on the apparent viscosity of XG-CC, GG-CC, and CS-CC composites. (**A**): XG-CC; (**B**): GG-CC; (**C**): CS-CC. The format of the legend in each sub-graph is “composite material name (pH, polysaccharide concentration)”.

The apparent viscosity of the XG-CC composite solution increased with higher XG concentrations (Figure 3A). As the pH increased, enhanced electrostatic interactions between molecules contributed to an upward trend in viscosity, which was the same result as in [34]. Even near the iso-electric point, the high-concentration XG-CC composite solution still showed relatively high viscosity, which may be attributed to the fact that the influence of XG on the viscosity of the composite solution was significantly greater than that of pH value. The main reason for this was that XG itself has superior pH value stability, which is very stable in acid-based solutions and has almost no variation in its viscosity within a large pH range.

When different concentrations of GG were added, the apparent viscosity of the GG-CC composite solution decreased with increases in shear rate (Figure 3B), showing a shear-thinning phenomenon and indicating that the low-acyl GG-CC composite system was a pseudoplastic fluid [35]. When the added amount of GG was less than 0.3%, the viscosity of the composite solution did not change significantly. With a high concentration, the viscosity of the GG-CC composite solution increased significantly with the increase in polysaccharides, indicating that the addition of GG had a certain synergic effect on the apparent viscosity. This was attributed to the fact that GG automatically aggregates with proteins through hydrogen bonding to form a stable three-dimensional network structure. According to a study by Moritaka et al. [36], the concentration of GG has a great influence on the rheological properties of the system, and the shear-thinning property increases with increases in the mass fraction of the colloid, which is consistent with the conclusions of this study. When pH ≥ 7, because pH > pI increases the number of negatively charged amino acid residues on the protein surface, GG and CC had the same charge, the electrostatic repulsive force between molecules increased, and their hydrophobic interaction was also enhanced. The hydromechanical radius of free GG molecules increased and the flow resistance increased, resulting in an upward trend in the viscosity of the composite solution [37].

As can be seen from Figure 3C, the apparent viscosity of the CS-CC composite solution showed an obvious upward trend with the increase in CS concentration. The apparent viscosity of the CS-CC composite system was higher than that of the blank group when the CS content was higher than 0.6%. The rheological curve of the viscosity and shear rate of the samples with high CS content had a higher initial viscosity, and the viscosity rapidly decreased under the low shear rate condition. This may have been related to the formation of new structures by the interaction between the polysaccharides and proteins [38]. Under the same concentration of CS, the apparent viscosity of the composite solution decreased first and then increased with increasing pH. When the pH value was different than the isoelectric point of CC (6.0), the electrostatic repulsion between CC and CS was enhanced, and the internal molecular structure was more compact, thus increasing the viscosity of the CS-CC composite solution [39]. Notably, the effect of pH on CS differed from its effect on XG and GG, likely because CS is a cationic polysaccharide, whereas XG and GG are anionic.

The distinct rheological behaviors of the three polysaccharide systems underscore the profound influence of molecular charge and structure on collagen interactions. The viscosity of the XG-CC and GG-CC composites was predominantly governed by the intrinsic polymer properties of the polysaccharides themselves. Both XG and GG, as high molecular weight anionic polysaccharides, impart high viscosity through chain entanglement and extensive hydration, forming a continuous network that dominates the system’s flow behavior, with collagen acting as a filler or co-solvent. In contrast, the CS-CC system exhibited a viscosity profile that was critically dependent on intermolecular associative interactions. The initial low viscosity across most pH levels reflected electrostatic repulsion between the cationic biopolymers. The observed viscosity peaks at specific pH-concentration combinations were not due to CS’s intrinsic viscosity, but rather to the formation of CS-CC associative networks driven by a balance of charge neutralization, hydrogen bonding, and hydrophobic interactions. This fundamental difference highlights that XG/GG enhance viscosity by diluting and structuring the aqueous phase, whereas CS enhances viscosity only when it can directly associate with collagen to form a percolated network.

### 3.4. Effect of pH on the Fluorescence Spectrum of PS–CC Composite

As CC is an incomplete protein lacking tryptophan, the intrinsic fluorescence derived from phenylalanine and tyrosine residues was utilized to monitor its conformational changes [40]. The polysaccharide itself can neither emit light in a natural state nor be excited to produce fluorescence, but it affects the exposure of luminescent groups in CC by reacting with groups between CC, which then affects the fluorescence intensity of the composite solution [41].

**Figure 4:**
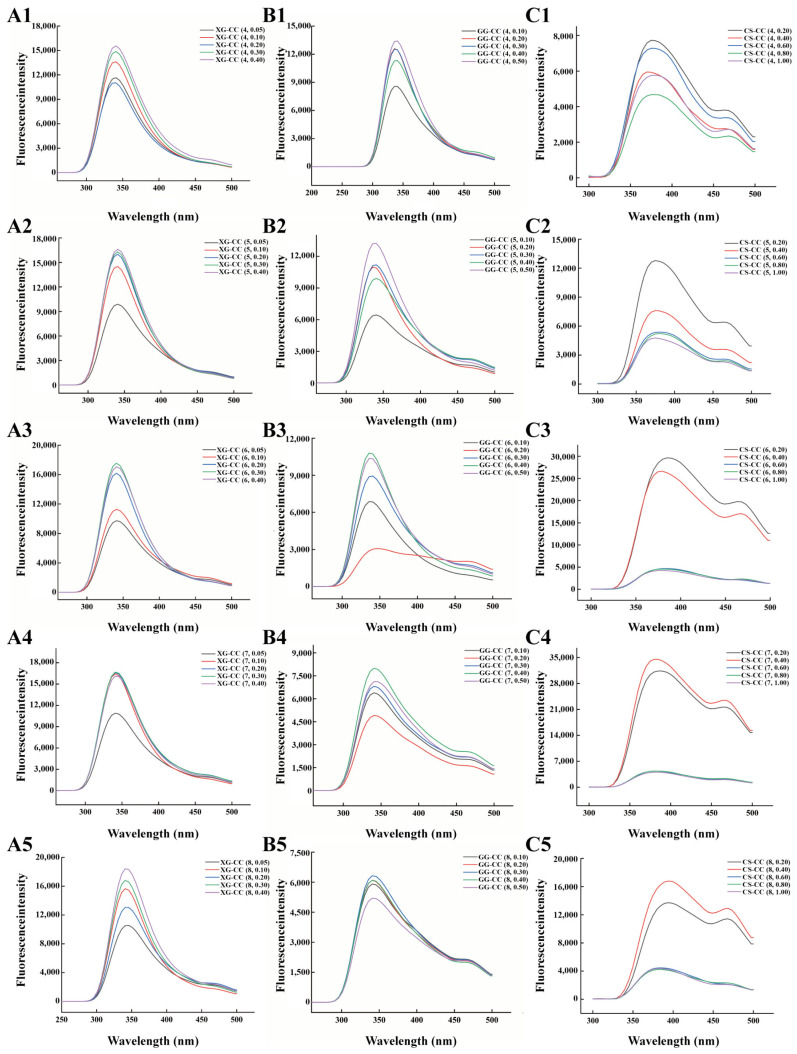
The effects of different pH values on the fluorescence of XG-CC, GG-CC, and CS-CC composites. (**A**): XG-CC; (**B**): GG-CC; (**C**): CS-CC. The format of the legend in each sub-graph is “composite material name (pH, polysaccharide concentration)”.

Figure 4A shows the effect of pH on the fluorescence spectrum of different added amounts of XG-CC composite solutions. When the amount of XG added was certain, the endogenous fluorescence intensity of the XG-CC composite solution at pH = 4 was the lowest. This shows that at low pH, there was a strong electrostatic interaction between the CC and XG molecules due to their opposite charges, and the bridging effect was not obvious, with CC forming a stable aggregate with XG and thereby embedding the partially exposed luminescent amino acid residues [42]. When the pH was greater than the isoelectric point of the protein, the fluorescence intensity of the XG-CC composite solution was greater, which was attributed to the fact that XG, as an anionic polysaccharide, has the same charge as CC. The two components having the same charge repelled and destroyed the internal hydrophobic structure of CC during their interaction, and more amino acid residues were thus exposed to an increasingly hydrophobic environment [43].

Figure 4B shows the effect of pH on the fluorescence spectrum of the GG-CC composite solutions with different added amounts. It can be seen from the figure that when the addition amount of gellan glue was low (≤0.2 wt%), the fluorescence intensity of the composite solution increased first and then decreased with the increase in pH, and the overall trend showed an increase; when the addition amount of gellan glue was greater than 0.2 wt%, the fluorescence intensity generally increased first and then decreased with the increase in the pH value of the composite solution. When the added amount of GG was certain, the number of amino acid residues with a positive charge on the CC surface increased at pH ≤ 5 and had the opposite charge to GG. Because the electrostatic interaction between the two was enhanced, the chromophore group was embedded within the molecule by the hydrophilic layer of the protein. When the pH approached the isoelectric point, the fluorescence intensity of the GG-CC composite solution tended to increase. The reason may have be that when the pH approached the pI of CC, the total surface charge of the protein was almost 0 and the internal hydrophobic groups were gradually exposed [44]. Chen Yang [45] also made a similar discovery when exploring the interaction between oat protein and GG. When the pH was close to the isoelectric point of oat protein, more chromophore groups were exposed, resulting in an increase in fluorescence intensity. At pH = 8, the change range of the fluorescence intensity of the GG-CC composite solution decreased slightly with the increase in the added amount of GG. The reason was that with the increase in the added amount of GG, more ions appeared in the solution, the probability of intermolecular collision increased, and the intermolecular charge interaction was enhanced, which led to a slight decrease in the fluorescence intensity of the GG-CC composite solution. In addition, the increase in viscosity also affected the stretch of the protein peptide chains and thus affected the change in fluorescence intensity [46].

It can be clearly seen from Figure 4C that the maximum emission wavelength of the luminescent amino acid of the protein had red shifted. This may have been because the increase in the amount of cationic polysaccharide had changed the steric hindrance between the protein particles, which affected the internal conformation of the CC [47]. The fluorescence intensity of the CS-CC composite solution increased significantly with the increase in CS concentration (0.2–0.8 wt%), indicating that more luminescent amino acid groups were exposed at this time. The notable enhancement in fluorescence intensity, coupled with the observed red shift, suggests that the tyrosine and phenylalanine residues were transferred into a more hydrophobic microenvironment upon complex formation. This is a strong indicator that hydrophobic interactions, alongside charge effects, contributed significantly to the association between CS and CC [48].

When the CS addition exceeded 0.4 wt%, the fluorescence intensity of the CS-CC composite solution reached its highest value at pH = 4 or 5. The reason for this trend may be attributed to the charge state of both biopolymers. At pH 4–5, which is below the isoelectric point (pI~6.0) of CC, the protein carries a net positive charge. CS, as a cationic polysaccharide, is also positively charged under acidic conditions. Despite the electrostatic repulsion between CC and CS, the high density of positive charges on CC might have enhanced the molecular collisions and increased the flexibility of the protein molecules, resulting in the ionization and exposure of more luminescent amino acids and thus an increase in fluorescence intensity [48].

In contrast, when the pH increased above 5, the fluorescence intensity decreased significantly. Near the isoelectric point of CC (pH~6.0), the net charge of the protein approached zero, leading to decreased solubility and possible condensation of the protein itself. Simultaneously, as the pH moved away from the pI, the strengthened charge interaction and non-covalent interaction between CS and CC may have caused more luminescent amino acids to be buried within the protein’s structural chain. Both mechanisms can lead to fluorescence quenching and a reduction in the observed fluorescence intensity of the CS-CC composite solution [48].

The comparative analysis of the fluorescence data reveals the interaction mechanisms between CC and the polysaccharides, dictated by their charge characteristics. For the anionic polysaccharides (XG and GG), the predominant mechanism at low pH is electrostatic attraction to the positively charged CC. This close association leads to the quenching of fluorescence intensity, as the tyrosine and phenylalanine residues become buried within the newly formed, insoluble complexes. In stark contrast, the interaction with the cationic polysaccharide (CS) is initially governed by electrostatic repulsion. However, at higher CS concentrations and a specific pH window (4–5), the repulsion is overcome, leading to complex formation through mechanisms like charge neutralization and polymer bridging. This association, surprisingly, results in a significant enhancement of fluorescence intensity. This key difference suggests that the CS-CC interaction induces a conformational change in the collagen molecule that exposes hydrophobic pockets, transferring the fluorophores to a less polar microenvironment—a clear signature of hydrophobic interactions playing a dominant role in the CS-CC system, which is distinct from the burial-driven quenching observed in the anionic systems.

### 3.5. Effect of pH on the Fourier Transform Infrared Spectroscopy of PS–CC Composite

**Figure 5:**
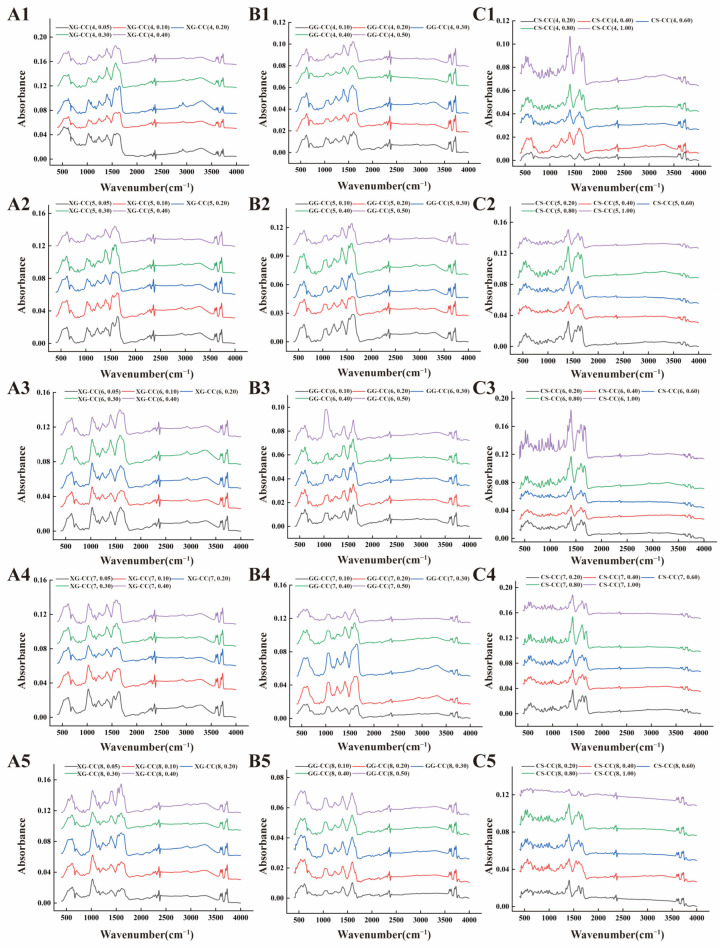
The effects of different pH values on the FT-IR of XG-CC, GG-CC, and CS-CC composites. (**A**): XG-CC; (**B**): GG-CC; (**C**): CS-CC. The format of the legend in each sub-graph is “composite material name (pH, polysaccharide concentration)”.

The addition of polysaccharides can affect the vibration of protein chemical bonds and functional groups, and thus infer the changes in protein internal molecular structure [49]. Therefore, the interaction between polysaccharide and CC can be studied by Fourier infrared spectroscopy. Figure 5A shows the effect of pH on the Fourier infrared spectrum of XG-CC composite. Under different pH conditions, the absorption peak of XG-CC composite appeared near 3700 cm^−1^, which was caused by the stretching vibration of O-H. Compared with single CC, the O-H absorption peak of XG moved to a higher wavelength range, indicating the interaction between XG and CC. In addition, with the increase in XG content, the peak absorption strength was enhanced and the peak deformation became sharp, indicating on the one hand that the hydrogen bond between CC and XG was strengthened, and on the other hand, the hydrogen bond between polysaccharide molecular chains was entangled [50]. There were three absorption peaks in the wavelength range of 1200~1700 cm^−1^, namely the amide Ⅰ band, Ⅱ band, and Ⅲ band. With the pH increased, the absorption peak of the amide Ⅰ band near 1600 cm^−1^ obviously existed, but the position of the absorption peak was red shifted, which was related to the stretching vibration of the C=O bond. When the hydroxyl group on XG, as a strong electron-absorbing group, was connected to the carbon atom on the protein amino acid, it fought for the electrons, thus reducing the vibration frequency of C=O and shifting the absorption peak to a low wave, indicating that the intermolecular hydrogen bond was affected by pH [51]. With the increase in pH, the peak area of the amide Ⅲ band in the wavelength range of 1320~1200 cm^−1^ decreased or even disappeared, which means that the increase in pH resulted in the disappearance of N-H deformation vibration among protein molecules, indicating that the electrostatic interaction between the -NH^3+^ group on the surface of CC and the -COO- group in XG weakened sharply. 

Figure 5B shows the effect of different pH on the Fourier infrared spectrum of GG-CC composite. As can be seen from the figure, there was an absorption peak near 3700 cm^−1^, where the absorption peak represents O-H stretching vibration. However, with the increase in the GG, the area of the absorption peak became larger, indicating that more hydrogen bonds formed between CC and the hydroxy group in GG. The absorption peak was relatively wide with the increase in pH value under the same GG addition, mainly because the intermolecular charge interaction under low pH (pH ≤ 5) was superior to the covalent bond interaction under high pH (pH > 5) [52]. Compared to the pH effect, the absorption peak intensity changed significantly with the GG concentration, indicating that the effect of pH on intermolecular hydrogen bonding was smaller than that of concentration, so the effect of hydrogen bonding was mainly related to the number of hydroxyl groups. GG-CC composite also has three absorption peaks of the amide Ⅰ band, Ⅱ band, and Ⅲ band between 1700 and 1200 cm^−1^. With the increase in pH value, the trend of absorption intensity in the amide Ⅰ and amide Ⅲ bands were like that XG-CC. It means that there was strong charge interaction and non-covalent interaction between GG and CC [53].

Figure 5C shows the Fourier infrared spectrum changed in the CS-CC composite under different pH conditions. The peak was not obvious, near the wavelength of 3500 cm^−1^. Since the characteristic absorption peak was the vibration peak of hydroxy-hydration, which was related to the formation of hydrogen bonds, it was indicated that when the amount of CS was between 0.4 and 0.8%, the hydrogen bonding force between CS-CC composite was not significant. Under the low addition of CS, a weak absorption peak near 3500 cm^−1^ appeared, demonstrating an interaction between the molecules due to their electrostatic attraction and hydrogen bonding. As the pH of the CS-CC composite increased, the characteristic peak of the amide Ⅱ band near 1500 cm^−1^ showed a redshift, indicating that the charge collision between CC and CS caused a stretching vibration of the N-H bond in the protein molecules, which changed the conformation of CC. It caused changes in the secondary structure of CC.

Collectively, the FTIR spectra across all three systems confirm that both hydrogen bonding (evidenced by shifts in the O–H/N–H stretching region around 3300 cm^−1^) and interactions involving the protein’s amide bonds (particularly Amide I and II) are fundamental to the formation of CC–polysaccharide composites. However, the specific manifestation and pH-dependence of these interactions are critically governed by the polysaccharide’s charge type. For the anionic polysaccharides (XG and GG), the most pronounced spectral changes (e.g., significant broadening of O–H peaks, notable shifts in Amide I) occurred under acidic conditions (pH < pI). This aligns with a mechanism dominated by electrostatically enhanced hydrogen bonding, where the initial attractive force brings the molecules into close proximity, facilitating strong hydrogen bond formation. In contrast, the cationic CS system exhibited weaker and more pH-sensitive hydrogen bonding signals. Its interaction with CC was strongest in a narrow pH window near the protein’s pI, where electrostatic repulsion is minimized. This suggests that for CS, hydrogen bonding operates not under the reinforcement of electrostatic attraction, but in competition with electrostatic repulsion, leading to a distinctly different interaction profile compared to the anionic polysaccharides.

### 3.6. Effect of NaCl on the Physical Appearance and Turbidity of PS–CC Composite

**Figure 6:**
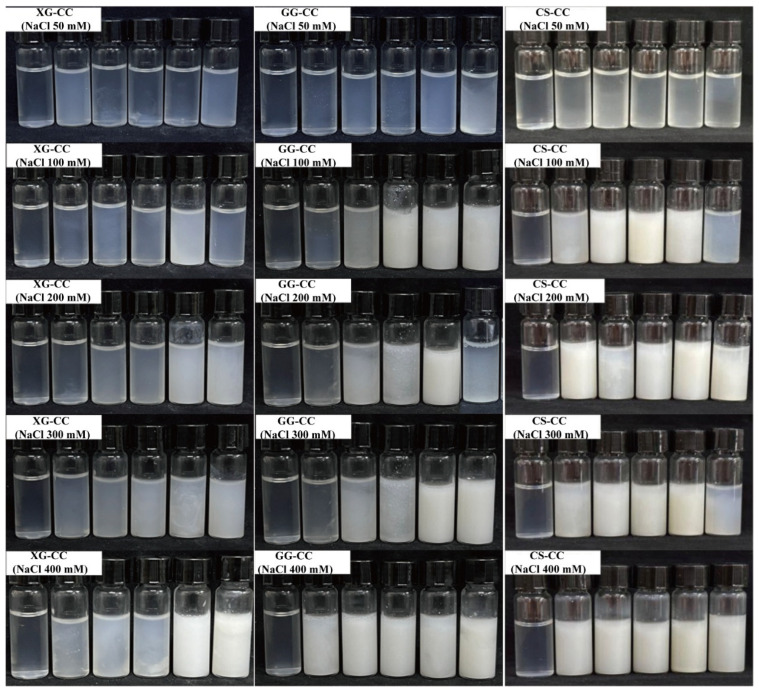
The effects of different NaCl concentrations on the physical appearance of XG-CC, GG-CC and CS-CC composites. There are six bottles in each subfigure, which were the control group and XG-CC, GG-CC, or CS-CC composites with five polysaccharides concentrations, increased in turn.

Figure 6 and Table 3 show the effects of different concentrations of NaCl on the physical appearance and turbidity of the XG-CC composite. As can be seen from Table 3, when the amount of XG added was low, the addition of NaCl further increased the compatibility of the system. When a high concentration (≥0.2%) of XG was added, the turbidity value of XG-CC gradually increased with the increase in NaCl concentration in the system. When NaCl concentration increased from 50 mM to 400 mM, the turbidity of composite of 0.3% XG group increased from 0.38 to 1.23. The reason was that CC was positively charged in an acidic environment, while XG was a negatively charged polysaccharide, and the molecules mainly interacted with electrostatics. When the amount of NaCl added was low, Cl^−^ and Na^+^ neutralized the charges on proteins and polysaccharides and weakened the interaction between the CC and XG molecules [54]. However, when a large amount of NaCl was added, a salting-out effect occurred in the CC, resulting in reduced protein solubility and precipitation and leading to an increase in the turbidity value of the XG-CC composite [55]. In addition, the opacity of XG itself would also increase the turbidity value of the composite [56].

Different concentrations of NaCl had a significant effect on the turbidity value of GG-CC composite. It can be seen from Figure 6 and Table 3 that the turbidity value of the GG-CC composite showed an increasing trend with the increase in GG. With the same amount of GG, when NaCl increased from 50 mM to 300 mM, the turbidity of GG-CC composite showed a positive correlation with the ion concentration. After a small amount of NaCl was added, the ions generated by the decomposition of NaCl could shield the charges on the surface of CC and GG. Therefore, the electrostatic interaction and charge interactions between molecules was weakened, and the turbidity value of GG-CC composite was low [48]. When the addition of GG exceeds 0.4%, the turbidity value of GG-CC composite was a little higher than that of the low addition group. While high GG content hindered the formation of the composite due to the increase in viscosity, the change trend in the turbidity value was not obvious.

The effects of NaCl on the turbidity of a CS-CC composite varies greatly with different CS added (Figure 6 and Table 3). When the amount of CS is low, the turbidity of the CS-CC composite increases first and then decreases with the increase in NaCl. At a higher CS concentration (higher than 0.4%), the addition of NaCl was prone to sedimentation [57]. Due to the fact that salt disrupts the electrostatic interaction between the protein and polysaccharide by shielding the charged reaction sites on the two biopolymers, the steric hindrance of the system decreased, resulting in a significant reduction in the electrostatic repulsion between particles and accelerated sedimentation, thus increasing the turbidity value [58]. This was also proved by Ding et al. [59] in their exploration of the phase behavior of CS/casein composite.

In summary, the response of turbidity to ionic strength underscores the primary role of electrostatic interactions. For the anionic polysaccharides (XG and GG), low NaCl concentrations screened the attractive forces with CC, reducing turbidity, while high concentrations promoted salting-out. In contrast, the CS-CC system, initially governed by electrostatic repulsion, exhibited a more complex non-monotonic response, where low-level charge shielding subtly modulated interactions before salting-out dominated at high ionic strength.

### 3.7. Effect of NaCl on the Apparent Viscosity of PS–CC Composite

**Figure 7:**
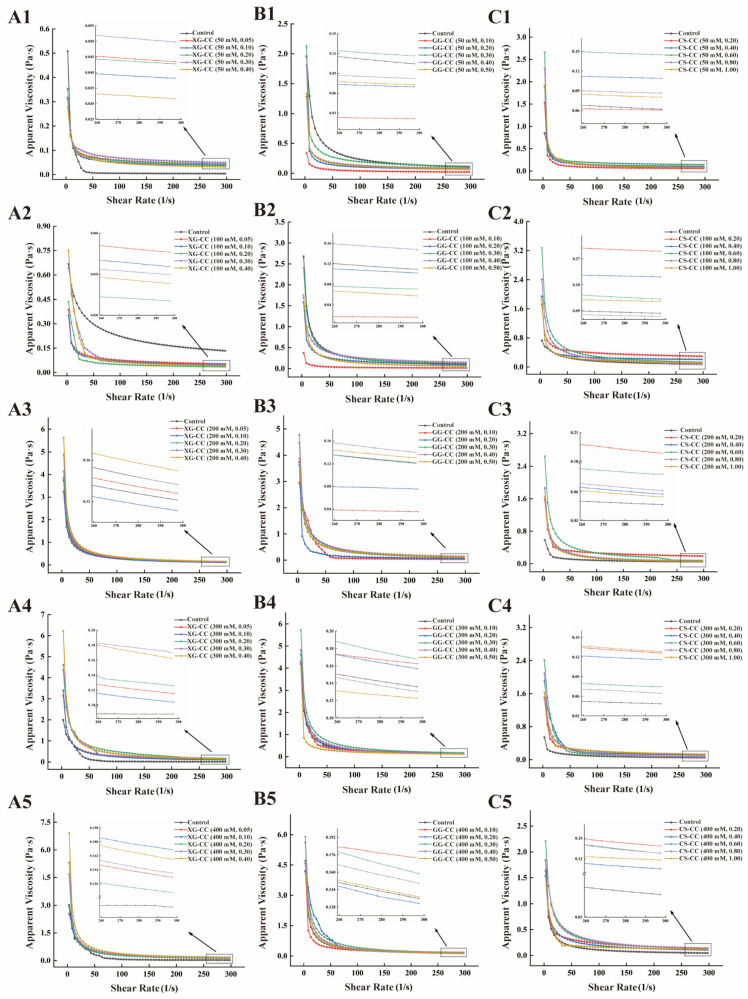
The effects of different NaCl concentrations on the apparent viscosity of XG-CC, GG-CC, and CS-CC composites. (**A**): XG-CC; (**B**): GG-CC; (**C**): CS-CC. The format of the legend in each sub-graph is “composite material name (NaCl concentration, polysaccharide concentration)”.

Figure 7A shows the influence of different NaCl concentrations on the apparent viscosity of the XG-CC composite solution as the shear rate changes. The apparent viscosity of the composite with different concentrations of NaCl decreased with increases in the shear rate. When the shear rate reached a certain level, the viscosity of the composite did not change significantly and tended to be almost stable, exhibiting typical shear-thinning behavior [60]. When the amount of XG was 0.05%, the addition of NaCl had a significant effect on the apparent viscosity of XG-CC composite. The apparent viscosity of solutions decreased first and then increased with the increase in NaCl, which may be due to the addition of Na^+^ shielding the negative ions on the surface of XG and weakening the electrostatic interaction between molecules. Na^+^ was not sufficient to completely shield the negatively charged XG, so the interaction between protein and polysaccharide was enhanced, and the solution was more prone to condensation. With the addition of XG, the viscosity of the solution showed an upward trend. When the amount of polysaccharide was constant, the increase in ion concentration was conducive to the formation of a network structure, which led to an increase in the viscosity of the composite. As reported by Baradossi et al., XG presents a disordered conformation in aqueous solution. When NaCl was added to the solution, the XG showed an ordered conformation due to the combination of Na^+^ as a crosslinking agent with the carboxyl group on its side chain, which increases the friction of ions in the solution and leads to the increase in the viscosity of XG-CC composite [61].

Figure 7B shows that under the same ionic strength, the viscosity of the GG-CC composite solution did not change significantly when the GG concentration was below 0.3%. However, when the concentration of GG remains unchanged, the viscosity changes significantly with the amount of NaCl added. When the ion concentration was low, the apparent viscosity of the solution was low, and the viscosity declined gently with the increase in shear rate. When NaCl concentration was in the range of 50–300 mM, the apparent viscosity of the solution increased, but the system viscosity no longer increased when the NaCl concentration exceeded 300 mM, and there was a downward trend. This may be attributed to the fact that the excessive Na^+^ may have hindered the interaction between GG and CC due to the competition between cations and may have even reduced the viscosity of the system. Therefore, the appropriate amount of GG and NaCl will increase the apparent viscosity of the composite and increase the stability of the system [62].

Figure 7C shows the trend of the apparent viscosity of the CS-CC composite solutions with different concentrations and shear rates under different amounts of added NaCl. The apparent viscosity of the CS-CC composite did not change significantly under different CS concentrations but was significantly different with the added ions. Overall, the apparent viscosity increased with the increase in ion concentration when NaCl was less than 200 mM. When the amount of NaCl added was more than 200 mM, the concentration of NaCl ion was negatively correlated with the apparent viscosity. NaCl could be decomposed into Na^+^ and Cl^−^ in the solution, and Cl^−^ in the solution system would shield the positive charge that existed on the CS chain. The change in the charge distribution of the amino acid side chain of the protein enhanced the interaction ability of the protein and CS, and the viscosity increased accordingly. However, when a large amount of NaCl was added, it could be decomposed into Na^+^ and would destroy the stability of the mixed system of protein and CS, and could even produce intermolecular aggregation, reducing the viscosity of the system. Therefore, the stability of the protein–polysaccharide composite could be regulated by ion concentration [63].

In summary, the ionic strength dictated the rheological behavior by modulating the dominant interaction in each system. For XG-CC, NaCl promoted an orderly conformation of XG chains, leading to a steady increase in viscosity. In contrast, for both GG-CC and CS-CC, excessive NaCl weakened the inter-molecular interactions (electrostatic and hydrogen bonding) essential for network integrity, resulting in a decline in viscosity after an initial increase. This highlights that an optimal ionic strength is required to maximize the rheological properties of polysaccharide–collagen composites, beyond which disruptive charge screening prevails.

### 3.8. Effect of Sodium Chloride on Fluorescence Spectrum of PS–CC Composite

**Figure 8:**
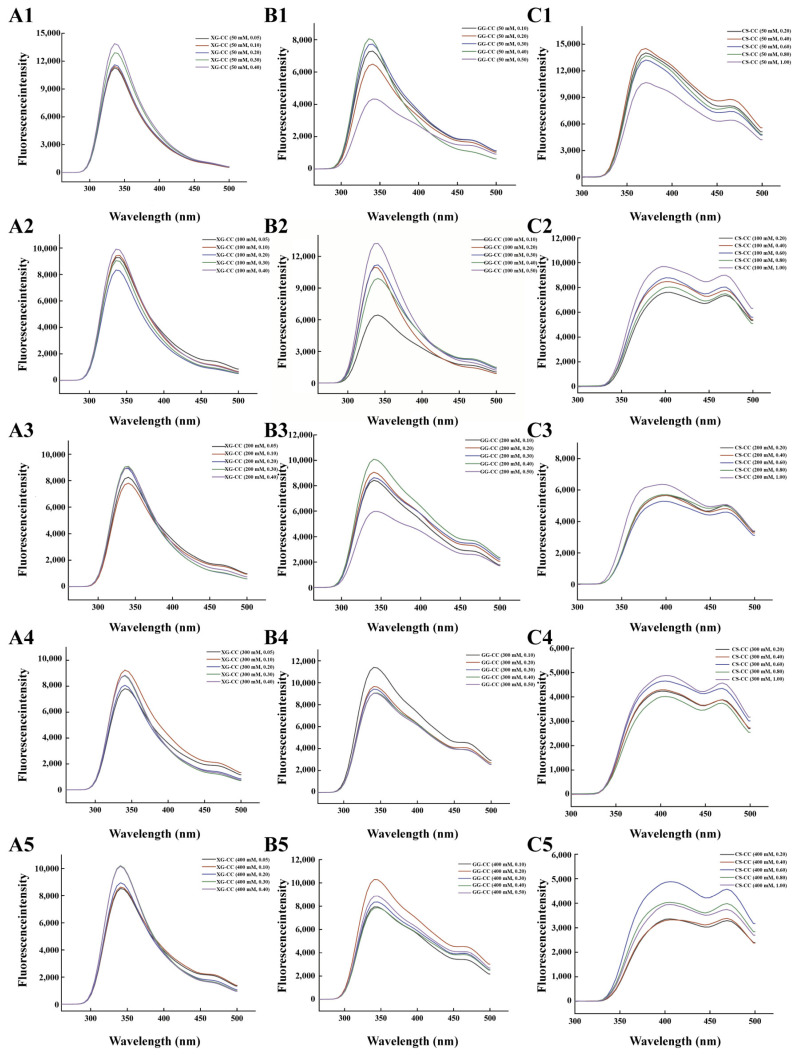
The effects of different NaCl concentrations on the fluorescence of XG-CC, GG-CC, and CS-CC composites. (**A**): XG-CC; (**B**): GG-CC; (**C**): CS-CC. The format of the legend in each sub-graph is “composite material name (NaCl concentration, polysaccharide concentration)”.

Figure 8A shows the effect of different added amounts of NaCl on the fluorescence spectrum of XG-CC composite solution. It can be seen from the analysis in the figure that when the added amount of XG was 0.05 wt%, the range of variation in the fluorescence intensity values of the XG-CC composite solution was large, while the range of variation in the fluorescence intensity values among the other four groups of composite solutions with different added amounts of XG was not significant. The reason may be that when the added amount of XG was low, the viscosity of the system was low and was not enough to hinder the formation of condensation between the protein and XG molecules. Thus, the luminescent amino acids of the protein were exposed, so the fluorescence intensity value was higher than that of other added amounts. When the amount of XG added was fixed, the fluorescence intensity of the XG-CC composite solution decreased first and then increased with the increase in NaCl added within the experimental range. With the increase in NaCl added, the strongest fluorescence position of the protein’s luminescent amino acid residues occurred in a red shift, indicating that under these conditions, the interaction between XG and CC increased the polarity of the microenvironment near the protein’s luminescent amino acid residues, weakened the hydrophobicity, and compacted the CC structure. This indicates that more luminescent amino acids were encapsulated in the protein, thus resulting in a decrease in the fluorescence intensity; when the addition amount of NaCl continued to increase, high concentrations of NaCl destroyed the hydration membrane of the protein, and more luminescent amino acids were exposed, thus causing an increase in the fluorescence intensity [64].

Figure 8B shows the effect of different NaCl concentrations on the fluorescence spectra of the GG-CC composite solutions with different amounts added. It can be clearly seen from the overall situation that the fluorescence intensity range of the GG-CC composite solution did not change significantly, indicating that GG had less influence on the GG-CC composite solution than NaCl. When the GG was added in the same amount, the fluorescence intensity of the composite solution increased first and then decreased with the increase in NaCl concentration. When the added amount of GG was less than 0.3 wt%, the NaCl concentration was in the range of 50–300 mM. With the increase in concentration, the fluorescence intensity value gradually increased. This was attributed to the fact that the addition of NaCl may have neutralized the positive charges on the CC surface, weakening the interaction between the gellan glue and CC, and more luminescent amino acids were excited, and subsequently exposed, so the fluorescence intensity value of the composite solution increased. When the added amount of GG exceeded 0.3 wt%, the fluorescence intensity of the composite solution with a low added amount of NaCl (≤200 mM) was obvious, which may have been related to the stability of GG. Sodium ions can form a connection with GG through water to form GG(-COO^−^)-Na^+^-H2O-Na^+^-GG(-COO^−^), thereby enhancing the electrostatic attraction of the carboxyl groups on GG and facilitating the aggregation of the stable double helix structure of GG. This weakens the charge interaction and electrostatic interaction between GG and CC, thus causing more luminescent amino acids to be exposed, thus causing an increase in the fluorescence intensity of the composite system [65].

Figure 8C shows the effect of NaCl concentration on the fluorescence spectra of the CS-CC composite solutions with different amounts of CS added. It can be clearly seen from the overall figure that the fluorescence intensity range of the CS-CC composite solution did not change significantly when the added amount of CS was greater than 0.4 wt%, indicating that when the added amount of CS was higher than a certain amount, the impact on the CS-CC composite solution was not obvious, indicating that there was an interaction between CS and CC that was caused by the increased exposure of the hydrophobic amino acids inside the protein under low concentrations of CS. When the added amount of CS was greater than 0.2 wt%, the fluorescence intensity of the composite solution first increased, then decreased, and then increased with the increase in NaCl concentration. This was attributed to the increase in viscosity under the condition of 50–200 mM NaCl concentration, which weakened the interaction between CS and CC to a certain extent; thus, more luminescent amino acids were excited and exposed, so the fluorescence intensity value of the composite solution increased [66]. When the amount of NaCl added was greater than 200 mM, the action of ions blocked the charged charges on the protein and CS was adsorbed on the surface of the protein, causing fluorescence quenching and resulting in a slight decrease in the fluorescence intensity. Continuing to increase the amount of NaCl added destroyed the hydration membrane on the protein surface, the structure became loose, and more hydrophobic amino acids were exposed, thus causing an increase in the fluorescence intensity of the composite system.

In summary, the fluorescence response of all systems to NaCl underscores its dual role in modulating protein conformation. At lower concentrations, ionic shielding weakened electrostatic interactions, leading to a partial unfolding and increased fluorescence intensity due to fluorophore exposure. At higher concentrations, the prevailing salting-out effect promotes protein condensation and aggregation, which can either quench fluorescence by burying residues or alter it via hydrophobic clustering. The extent of these changes is system-specific, being most pronounced in complexes where electrostatic forces initially play a dominant role.

### 3.9. Effect of NaCl on the Fourier Transform Infrared Spectroscopy of PS–CC Composite

**Figure 9:**
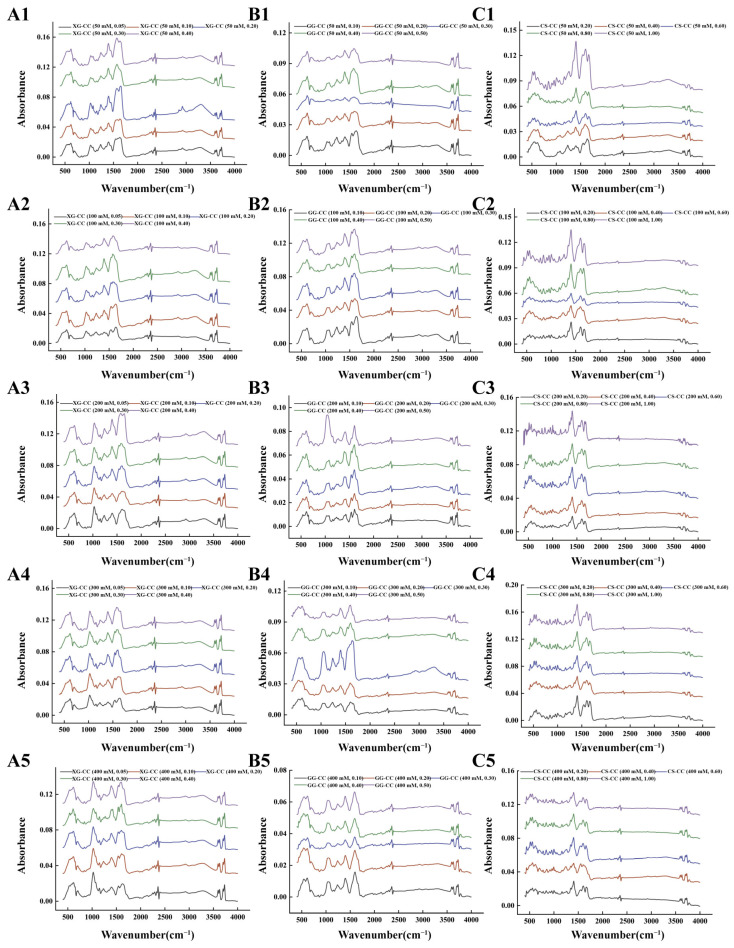
The effects of different NaCl concentrations on the FT-IR of XG-CC, GG-CC, and CS-CC composites. (**A**): XG-CC; (**B**): GG-CC; (**C**): CS-CC. The format of the legend in each sub-graph is “composite material name (NaCl concentration, polysaccharide concentration)”.

The infrared spectrum of the composite showed that the characteristic peak of the XG-CC composite was detected at 3600 cm^−1^ under different added amounts of NaCl (Figure 9A), and that this characteristic peak was caused by O-H stretching vibration. However, with the increase in XG content, the absorption peak changed insignificantly, meaning that the XG content had little effect on the hydrogen bonding between CC and XG. In the range of 1200–1700 cm^−1^, there were amide Ⅰ bands, Ⅱ bands, and Ⅲ bands, and the generation of these bands was related to C=O stretching vibration and N-H bending vibration. With the increase in NaCl, the absorption peak deformation was sharp, and the peak area was larger, which indicated that the addition of a high concentration of NaCl shielded the charge on the protein surface, and the electrostatic repulsion was enhanced. In the wave number range of 1335~1200 cm^−1^, the peak area of the amide Ⅲ band decreased or even disappeared, and the change in the absorption peak indicated the electrostatic interaction between the carboxyl group of XG and the amino group of the protein. In addition, the formation of electrostatic interactions also promoted a weak shift in the amide I band, and that slight change had also been observed in previous Raman spectroscopy papers [67]. An obvious absorption peak at wavelength 1000 cm^−1^ was observed, which may have been caused by the acetyl of XG [68].

Figure 9B shows the effect of different NaCl concentrations on the Fourier infrared spectroscopy of the GG-CC composite. As can be seen from the infrared spectra, the characteristic peaks were detected at 3600 cm^−1^. The results showed that there were intermolecular and intramolecular hydroxyl stretching vibrations of the GG-CC composite, indicating the existence of hydrogen bonding between GG and CC. The change in amide bond peak strength can be used as a characterization of the composite aggregation. The GG-CC composite had amide Ⅰ, Ⅱ, and Ⅲ bands, which were mainly related to the functional group change vibrations, such as C-N tensile vibration, C-H bending vibration, N-H bending vibration, and C=O tensile vibration [69]. The observed peak area of 1660–1620 cm^−1^ decreased with the increase in NaCl. The peak change here was attributed to the conjugating effect of the addition of Na^+^, which weakened the double bond properties in the original group, thus reducing the force constant and the absorption frequency [70].

The obvious characteristic peaks of the CS-CC composite under different conditions can be detected (Figure 9C), which proves that covalent binding formed between CS and CC. In the Fourier transform infrared spectrum of the CS-CC composite, the O-H stretching vibration absorption peak was at 3500 cm^−1^, and the C=O stretching vibration peak was at 1600–1700 cm^−1^ of the amide I band of CC. The C-H bending vibration absorption peak at 1400 cm^−1^ was obvious in the composite. In addition, under the condition of a low added amount of CS, the addition of NaCl increased the strength of the characteristic absorption bands of the amide I and II bands, and displaced the amide I band to a higher wave number, which means that more a disordered conformation was obtained with the increase in NaCl [71]. The slight increase in the strength of some amide bands in the 1500–1700 cm^−1^ range may have been related to the amino and carbonyl groups, demonstrating that these groups interact mainly through electrostatic interactions and hydrogen bonds. It can be inferred that the subtle differences may have been related to the slight interaction between the amino group of CS and the negatively charged carboxyl group of CC in the composite, and that these newly formed hydrogen bonds and intermolecular interactions both interfered with the wavelength range of the absorption peak and the vibratory absorbance of the functional group [72].

In summary, the FTIR spectra under varying ionic strengths reveal a common governing mechanism: the addition of NaCl primarily shields electrostatic interactions and disrupts the hydrogen-bonding network within the composites, as evidenced by the altered O–H stretching and amide band intensities. The extent of these changes, however, varies with the polysaccharide type, reflecting their differing initial reliance on electrostatic forces for complexation with collagen.

## 4. Conclusions

This study elucidated the interaction mechanisms and solution behaviors of cowhide collagen (CC) with three representative polysaccharides—xanthan gum (XG), gellan gum (GG), and chitosan (CS)—under various environmental conditions. The findings demonstrated that polysaccharide type and concentration exerted predominant influences on the physicochemical and rheological characteristics of the composite systems, surpassing the effects of pH and ionic strength. Anionic polysaccharides (XG and GG) primarily interacted with CC through hydrogen bonding and electrostatic attraction, while hydrophobic interactions became particularly relevant at pH values above the pI of CC. Conversely, the cationic CS exhibited distinct pH-responsive behaviors due to electrostatic repulsion, with aggregation at intermediate pH being driven by a combination of charge neutralization and hydrophobic effects. These insights provide a scientific foundation for tailoring collagen–polysaccharide interactions to design functional food systems with improved texture and stability. Specifically, XG–CC and GG–CC composites exhibit potential for use in collagen-enriched beverages, soups, sauces, and gel-based desserts, where controlled viscosity and network formation are desirable. Meanwhile, the pH-sensitive CS–CC complex shows promise in edible films, bio-based coatings, and packaging materials that require tunable permeability and mechanical resilience. The study thus supports the sustainable utilization of bovine by-products, contributing to waste valorization and the development of high-value functional ingredients in the food industry.

Nevertheless, this study primarily focused on steady-state rheological and spectroscopic characterizations, whereas dynamic properties such as gelation kinetics, emulsifying behavior, and long-term stability have yet to be systematically investigated. Future research should integrate these aspects through multi-scale structural analysis, simulation of processing conditions, and sensory evaluation of formulated products, to better translate the molecular-level mechanisms into practical applications. Such investigations will further advance the rational design and industrial deployment of collagen–polysaccharide composites in next-generation functional foods and biodegradable materials.

## Figures and Tables

**Table 1:** Amino acid composition of cowhide collagen extracted by EEM and UEEM.

No.	Amino Acid Type	Amino Acid Content (%)	
		EE	UEE
1	Aspartic acid	0.02 ± 0.01 ^a^	0.04 ± 0.01 ^a^
2	Glutamic acid	1.36 ± 0.13 ^b^	2.43 ± 0.09 ^a^
3	Serine	2.95 ± 0.22 ^b^	3.61 ± 0.17 ^a^
4	Glycine	29.96 ± 0.31 ^a^	27.14 ± 0.19 ^b^
5	Histidine	1.10 ± 0.06 ^a^	0.93 ± 0.04 ^b^
6	Arginine	7.49 ± 0.08 ^a^	6.97 ± 0.07 ^b^
7	Threonine	1.92 ± 0.05 ^a^	1.09 ± 0.03 ^b^
8	Alanine	10.16 ± 0.15 ^a^	8.22 ± 0.06 ^b^
9	Proline	29.39 ± 0.23 ^b^	35.96 ± 0.26 ^a^
10	Tyrosine	2.82 ± 0.02 ^a^	2.14 ± 0.01 ^b^
11	Valine	0.28 ± 0.06 ^a^	0.24 ± 0.03 ^a^
12	Methionine	2.96 ± 0.03 ^a^	2.51 ± 0.01 ^b^
13	Cystine	0.13 ± 0.01 ^b^	0.46 ± 0.02 ^a^
14	Isoleucine	1.54 ± 0.01 ^a^	1.44 ± 0.03 ^b^
15	Leucine	3.01 ± 0.03 ^a^	2.64 ± 0.02 ^b^
16	Phenylalanine	1.93 ± 0.02 ^b^	2.00 ± 0.01 ^a^
17	Lysine	2.98 ± 0.04 ^a^	2.16 ± 0.03 ^b^

Data are expressed as the mean ± standard deviation (S.D.) (*n* = 3). Different lowercase letters (a, b) indicate significant differences among different samples (*p* < 0.05).

**Table 2:** Effects of different pH values on the turbidity of XG-CC, GG-CC, and CS-CC composites.

pH	Additive Amount of Xanthan Gum					Additive Amount of Gellan Gum					Additive Amount of Chitosan				
	0.05%	0.1%	0.2%	0.3%	0.4%	0.1%	0.2%	0.3%	0.4%	0.5%	0.2%	0.4%	0.6%	0.8%	1%
4	0.19 ± 0.01 ^a^	0.77 ± 0.03 ^a^	0.89 ± 0.12 ^a^	1.03 ± 0.17 ^a^	1.12 ± 0.09 ^a^	0.47 ± 0.03 ^a^	0.69 ± 0.04 ^a^	0.88 ± 0.01 ^a^	1.13 ± 0.12 ^c^	1.23 ± 0.20 ^a^	0.19 ± 0.01 ^c^	0.21 ± 0.01 ^a^	1.56 ± 0.01 ^a^	1.39 ± 0.06 ^a^	2.27 ± 0.02 ^b^
5	0.15 ± 0.01 ^b^	0.22 ± 0.01 ^b^	0.87 ± 0.03 ^a^	0.27 ± 0.02 ^c^	0.46 ± 0.02 ^b^	0.16 ± 0.01 ^b^	0.33 ± 0.01 ^b^	0.33 ± 0.03 ^d^	1.91 ± 0.03 ^b^	2.07 ± 0.02 ^b^	0.24 ± 0.02 ^a^	0.21 ± 0.03 ^b c^	1.25 ± 0.02 ^b^	1.37 ± 0.02 ^a^	2.32 ± 0.02 ^a^
6	0.13 ± 0.01 ^c^	0.19 ± 0.03 ^b^	0.36 ± 0.05 ^b^	0.23 ± 0.02 ^c^	0.54 ± 0.03 ^b^	0.18 ± 0.01 ^b^	0.36 ± 0.07 ^b^	0.39 ± 0.01 ^c^	2.20 ± 0.13 ^a^	2.14 ± 0.01 ^b^	0.17 ± 0.01 ^c^	0.22 ± 0.02 ^b c^	0.11 ± 0.03 ^c^	0.12 ± 0.01 ^c^	0.10 ± 0.01 ^d^
7	0.13 ± 0.01 ^b c^	0.21 ± 0.03 ^b^	0.31 ± 0.02 ^b^	0.43 ± 0.01 ^b^	0.49 ± 0.04 ^b^	0.19 ± 0.01 ^b^	0.29 ± 0.01 ^b^	0.34 ± 0.04 ^d^	2.23 ± 0.05 ^a^	2.33 ± 0.06 ^a^	0.15 ± 0.01 ^d^	0.14 ± 0.01 ^c^	0.10 ± 0.06 ^c^	0.12 ± 0.01 ^c^	0.11 ± 0.01 ^d^
8	0.17 ± 0.01 ^a^	0.23 ± 0.01 ^b^	0.25 ± 0.02 ^b^	0.46 ± 0.03 ^b^	0.46 ± 0.02 ^b^	0.15 ± 0.03 ^b^	0.22 ± 0.01 ^c^	0.44 ± 0.03 ^b^	2.10 ± 0.08 ^a^	2.20 ± 0.01 ^a b^	0.21 ± 0.01 ^b^	0.10 ± 0.01 ^b^	0.17 ± 0.05 ^c^	0.18 ± 0.01 ^b^	0.17 ± 0.01 ^c^

Data are expressed as the mean ± standard deviation (S.D.) (*n* = 3). Different lowercase letters (a, b, c, d) indicate significant differences between samples with different pH (*p* < 0.05).

**Table 3:** Effects of different NaCl concentrations on the turbidity of XG-CC, GG-CC, and CS-CC composites.

NaCl Concentrations (mM)	Additive Amount of Xanthan Gum					Additive Amount of Gellan Gum					Additive Amount of Chitosan				
	0.05%	0.1%	0.2%	0.3%	0.4%	0.1%	0.2%	0.3%	0.4%	0.5%	0.2%	0.4%	0.6%	0.8%	1%
50	0.25 ± 0.01 ^a^	0.18 ± 0.01 ^c^	0.16 ± 0.01 ^d^	0.36 ± 0.05 ^e^	0.46 ± 0.01 ^e^	0.12 ± 0.03 ^c^	0.19 ± 0.01 ^c^	0.35 ± 0.02 ^d^	0.65 ± 0.03 ^d^	1.70 ± 0.01 ^c^	0.45 ± 0.04 ^c^	1.48 ± 0.03 ^c^	2.02 ± 0.01 ^a^	1.94 ± 0.03 ^b^	2.07 ± 0.01 ^a^
100	0.17 ± 0.01 ^b^	0.22 ± 0.02 ^b^	0.26 ± 0.03 ^d^	0.54 ± 0.01 ^d^	0.60 ± 0.04 ^d^	0.27 ± 0.04 ^b^	0.58 ± 0.01 ^b^	1.87 ± 0.01 ^b^	1.75 ± 0.03 ^c^	1.71 ± 0.06 ^c^	0.54 ± 0.02 ^a^	1.62 ± 0.02 ^b^	1.91 ± 0.08 ^b^	2.07 ± 0.01 ^a^	2.00 ± 0.06 ^a^
200	0.13 ± 0.05 ^b^	0.17 ± 0.01 ^c^	0.51 ± 0.01 ^c^	0.67 ± 0.02 ^c^	0.74 ± 0.04 ^c^	0.14 ± 0.01 ^c^	1.36 ± 0.02 ^a^	1.89 ± 0.01 ^b^	1.91 ± 0.01 ^b^	1.84 ± 0.01 ^b^	0.49 ± 0.01 ^b c^	1.83 ± 0.04 ^a^	1.91 ± 0.01 ^b^	2.05 ± 0.02 ^a^	2.04 ± 0.06 ^a^
300	0.24 ± 0.05 ^a^	0.47 ± 0.04 ^a^	0.87 ± 0.05 ^b^	0.97 ± 0.02 ^b^	1.18 ± 0.04 ^b^	0.25 ± 0.01 ^b^	1.35 ± 0.01 ^a^	1.97 ± 0.02 ^a^	1.97 ± 0.01 ^a^	1.90 ± 0.01 ^a^	0.53 ± 0.02 ^a b^	1.69 ± 0.04 ^b^	2.05 ± 0.05 ^a^	2.06 ± 0.01 ^a^	2.06 ± 0.04 ^a^
400	0.27 ± 0.01 ^a^	0.25 ± 0.01 ^b^	1.24 ± 0.17 ^a^	1.24 ± 0.04 ^a^	1.35 ± 0.04 ^a^	0.43 ± 0.07 ^a^	1.36 ± 0.03 ^a^	1.74 ± 0.05 ^c^	1.72 ± 0.02 ^c^	1.66 ± 0.02 ^c^	0.32 ± 0.01 ^d^	1.17 ± 0.09 ^c^	2.05 ± 0.05 ^a^	1.67 ± 0.01 ^c^	1.85 ± 0.02 ^b^

Data are expressed as the mean ± standard deviation (S.D.) (*n* = 3). Different lowercase letters (a, b, c, d, e) indicate significant differences between samples with different NaCl concentrations (*p* < 0.05).

## Data Availability

The original contributions presented in this study are included in the article/Supplementary Materials. Further inquiries can be directed to the corresponding authors.

## Supplementary Materials

The following supporting information can be downloaded at: https://www.mdpi.com/article/10.3390/foods14234107/s1, Figure S1: Extraction process of CC; Figure S2: Effect of pH and NaCl concentration on the Solubility of Cowhide Collagen; Table S1: Functional characteristic of both EE and UEE.
