# Supplementary material for: Comparative Interaction Mechanisms and Solution Behavior of Cowhide Collagen with Xanthan Gum, Gellan Gum, and Chitosan Under Variable Environmental Conditions
Source: Foods. 2025 Nov 29;14(23):4107. doi: 10.3390/foods14234107 (PMC12692376; doi:10.3390/foods14234107)
Supplement: Supplementary file 1 [file foods-14-04107-s001.zip › foods-3979898-supplementary.pdf]

Supplementary Material Description

The extraction of cowhide collagen (CC) commenced with a series of preparatory steps: fresh cowhide was defatted, non-collagen proteins were removed, and the material was cold-stored, thawed, high-speed chopped, and subjected to acid-swelling pretreatment.

Following pretreatment, collagen was extracted via two distinct protocols: **Conventional Enzymatic Extraction Method (EEM):** The homogenized hide was directly hydrolyzed with pepsin (105 U/g collagen) at 37°C for 8 hours. **Ultrasonic-Assisted Enzymatic Extraction Method (UEEM):** The homogenate mixed with pepsin was first treated with ultrasound (161 W, 64 minutes), followed by enzymatic hydrolysis at 37°C for the remainder of the total 8-hour duration.

Both methods subsequently converged for the final purification steps: salt precipitation, dialysis, and freeze-drying, yielding the final collagen products designated as EE and UEE, respectively.

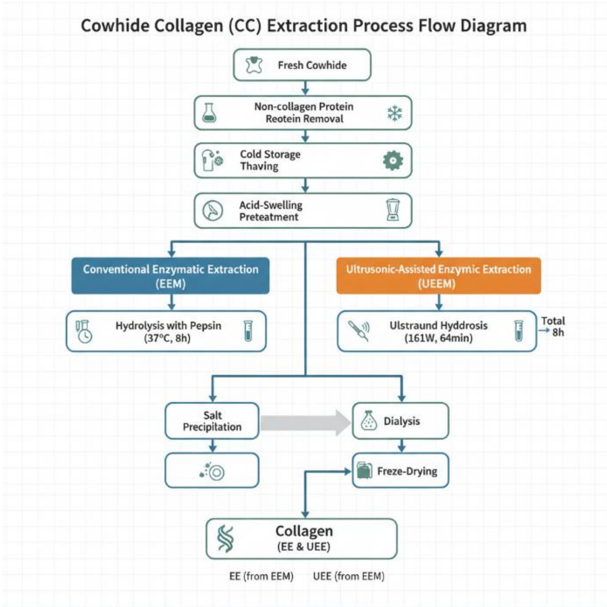

Figure S1 Extraction process of CC

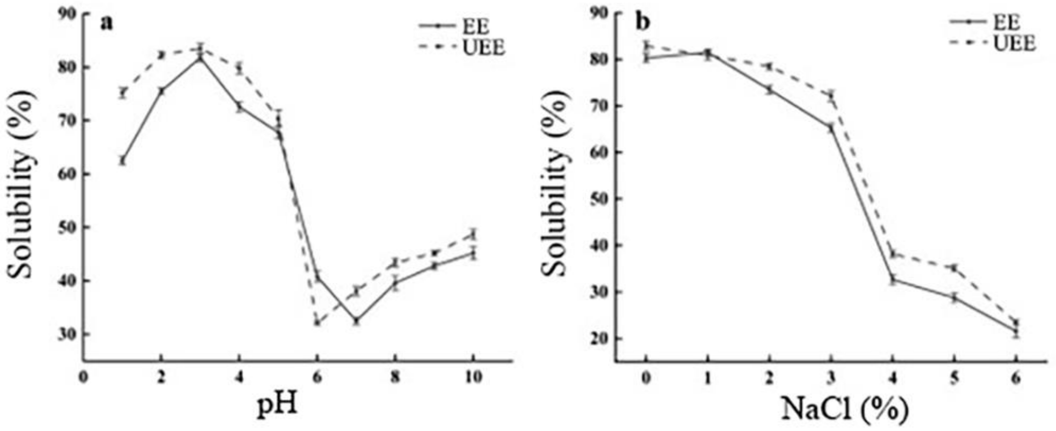

Fig. S2 Effect of pH and NaCl concentration on the Solubility of Cowhide Collagen

Figure S2 illustrates the effects of pH and NaCl concentration on the solubility of cowhide collagen. Collagen solubility is a critical parameter determining extraction efficiency, stability, and functionality in subsequent applications. As shown, solubility was highest under mildly acidic conditions (pH 3–4) and decreased sharply near the isoelectric point (pH ≈ 6), where electrostatic repulsion among protein molecules was minimized and aggregation occurred. Increasing NaCl concentration initially promoted solubility due to electrostatic shielding effects but resulted in a “salting-out” phenomenon at higher concentrations (>3%). These findings provide a physicochemical basis for optimizing extraction and application conditions to maintain collagen stability and solubility.

Tab. S1 Functional characteristic of both EE and UEE

Functional characteristic	Unit	EE	UEE
Oil holding capacity	mL/g	17.01±0.24^{b}	28.13±0.45^{a}
Emulsifying activity index (EAI)	m^2/g	8.35±0.19^{b}	14.68±0.27^{a}
Emulsification stability index (ESI)	Min	25.85±0.97^{b}	36.32±1.67^{a}
Foamability (%)	%	32.59±1.25^{b}	56.24±2.01^{a}
Foaming stability (%)	%	28.32±2.35^{b}	42.47±1.94^{a}

Table S1 compares the functional characteristics of enzyme extract (EE) and ultrasound-assisted enzymatic extract (UEE) of cowhide collagen. Functional indices, including oil holding capacity, emulsifying activity index (EAI), emulsifying stability index (ESI), foaming capacity, and foam stability, were measured to evaluate the applicability of collagen in food systems. UEE exhibited markedly enhanced performance across all parameters compared with EE ($p < 0.05$). Specifically, UEE showed a significantly higher oil holding capacity (28.13 ± 0.45 mL/g) than EE (17.01 ± 0.24 mL/g), suggesting improved hydrophobic interactions and greater molecular flexibility. Similarly, the EAI and ESI of UEE (14.68 ± 0.27 m^2/g and 36.32 ± 1.67 min, respectively) were substantially higher than those of EE (8.35 ± 0.19 m^2/g and 25.85 ± 0.97 min), indicating superior interfacial adsorption and emulsion stabilization properties. Furthermore, UEE demonstrated enhanced foaming capacity (56.24%) and foam stability (42.47%) compared with EE (32.59% and 28.32%, respectively), implying that ultrasound pretreatment facilitated the partial unfolding of collagen molecules, exposing both hydrophilic and hydrophobic domains that promote air–water interface activity. The improved functional performance of UEE can be attributed to the synergistic action of ultrasound and enzymatic hydrolysis. Acoustic cavitation generated during ultrasonic treatment disrupts the tight fibrillar structure of collagen, thereby increasing enzyme accessibility to peptide bonds. This process enhances

hydrolysis efficiency and promotes molecular disaggregation without destroying the triple-helical conformation of type I collagen, as verified by SDS–PAGE and FTIR analyses in the main text. Consequently, the UEE method yields collagen with more homogeneous molecular weight distribution, higher surface activity, and greater water retention ability, all of which are beneficial for forming stable colloidal systems.

The improved solubility, emulsifying performance, and foam-forming ability of UEE-derived collagen provide substantial advantages for its use in food formulations requiring interfacial stabilization or textural modification. Based on these findings, the collagen extracted using the UEE method was selected as the model substrate for subsequent experiments examining interactions between cowhide collagen and polysaccharides (xanthan gum, gellan gum, and chitosan). The superior functional attributes of UEE—particularly its enhanced emulsification and foaming performance—make it an ideal candidate for exploring the rheological, structural, and interfacial behaviors of collagen–polysaccharide composite systems under various pH and ionic conditions.

In summary, the supplementary data clearly demonstrate that ultrasound-assisted enzymatic extraction (UEE) significantly improves the structural integrity and functional properties of cowhide collagen compared with conventional enzymatic extraction (EE). These enhancements justify the selection of UEE-derived collagen for all subsequent analyses of collagen–polysaccharide interactions in this study, providing a reliable foundation for understanding and optimizing its potential applications in food systems.
